# Scanning speed phenomenon in contact-resonance atomic force microscopy

**DOI:** 10.3762/bjnano.9.87

**Published:** 2018-03-21

**Authors:** Christopher C Glover, Jason P Killgore, Ryan C Tung

**Affiliations:** 1 Department of Mechanical Engineering, University of Nevada, Reno, 1664 N Virginia St, Reno, NV 89557, USA; 2 National Institute of Standards and Technology, Applied Chemicals and Materials Division, 325 Broadway, Boulder, CO 80305, USA

**Keywords:** atomic force microscope, contact resonance, liquid, phenomenon, scan speed

## Abstract

This work presents data confirming the existence of a scan speed related phenomenon in contact-mode atomic force microscopy (AFM). Specifically, contact-resonance spectroscopy is used to interrogate this phenomenon. Above a critical scan speed, a monotonic decrease in the recorded contact-resonance frequency is observed with increasing scan speed. Proper characterization and understanding of this phenomenon is necessary to conduct accurate quantitative imaging using contact-resonance AFM, and other contact-mode AFM techniques, at higher scan speeds. A squeeze film hydrodynamic theory is proposed to explain this phenomenon, and model predictions are compared against the experimental data.

## Introduction

With the rise in popularity of simultaneous topographic imaging and material property quantification in atomic force microscopy (AFM) techniques, there exists a myriad of unexplained measurement phenomena caused by mechanical interactions between the scanning AFM tip and the material sample under test. In this article, we show how the velocity at which the tip is swept across the sample surface can affect the accuracy of the output data of AFM experiments. We focus exclusively on contact-mode AFM techniques. In particular, we study these phenomena using contact-resonance (CR) AFM techniques [1]. CR has been chosen in this study because it operates in the linear repulsive region of the tip–sample interaction, in permanent contact with the surface, alleviating the complicated effects introduced by liquid environments and nonlinear tip–sample interaction forces. CR methods can measure surface elastic [2], viscoelastic [3], electromechanical [4], and chemical properties [5]. For mechanical properties, the methods are well understood, producing highly accurate quantitative measurements at the nanoscale [6].

The effect of scan velocity, with regards to the dynamic behavior of the tip–sample interaction in AFM, has been largely ignored [7–9]. Enhancement of scan speeds in AFM is a rich and vibrant area of research, but to date most works have dealt with increasing the scan resolution [10], increasing the frequency bandwidth of the AFM electronics [11], and eliminating scanning hysteresis [12]. As the field of AFM advances, so too does the speed at which dynamic scanning occurs. Butt et al. [13] predicted the theoretical scanning speed limit, in terms of the maximum achievable resolution, and other researchers such as Bosse et al. [14] have created methods to more accurately measure relevant system parameters, such as the friction coefficient, at higher scan speeds. What is not fully understood is the effect that dynamic scanning, coupled with the mechanical interaction of the test sample, has on the AFM system at ever increasing scan speeds. Accurate AFM measurements are impossible at higher scan speeds without explicit understanding of these new scan speed phenomena.

Recently, there has been some mention of scan-speed effects in the literature. Picco et al. [15] reported an apparent decrease in forces applied to the measured sample when using high-speed contact mode AFM versus conventional-speed contact mode AFM. Additionally, they measured the lateral forces as a function of scan speed and reported a phenomenological change in the observed forces when the scan speed was higher than a critical speed. Picco et al. [15] proposed two possible mechanisms for this observed effect: superlubricity and scan-speed dependence on the no-slip fluid boundary condition. Scan-speed dependence has also been observed in contact-resonance spectroscopy experiments. Killgore et al. [3] reported a scan-speed dependence of the measured CR frequencies of an AFM cantilever. Above a critical speed, CR frequency and quality factor decreased with increasing scan speed. However, in that work, the sample surface was polymeric, and thus viscoelastic effects could not be ruled out as a root cause of the observed trend. It may be possible to avoid the in-contact scanning-speed phenomenon altogether through specialized AFM scanning modes, such as intermittent-contact scanning modes. However, this option may be unavailable to researchers due to various experimental and theoretical constraints. Additionally, such effects cannot be entirely ruled out for lower-frequency (e.g., sub-resonance) intermittent-contact methods. In this article, we present experimental results on a model non-viscoelastic hydrophilic sample to show that hydrodynamic stiffness of an adsorbed water layer is a plausible explanation for scan speed-induced changes in the mechanical coupling of tip and sample.

## Theory

**Figure 1:**
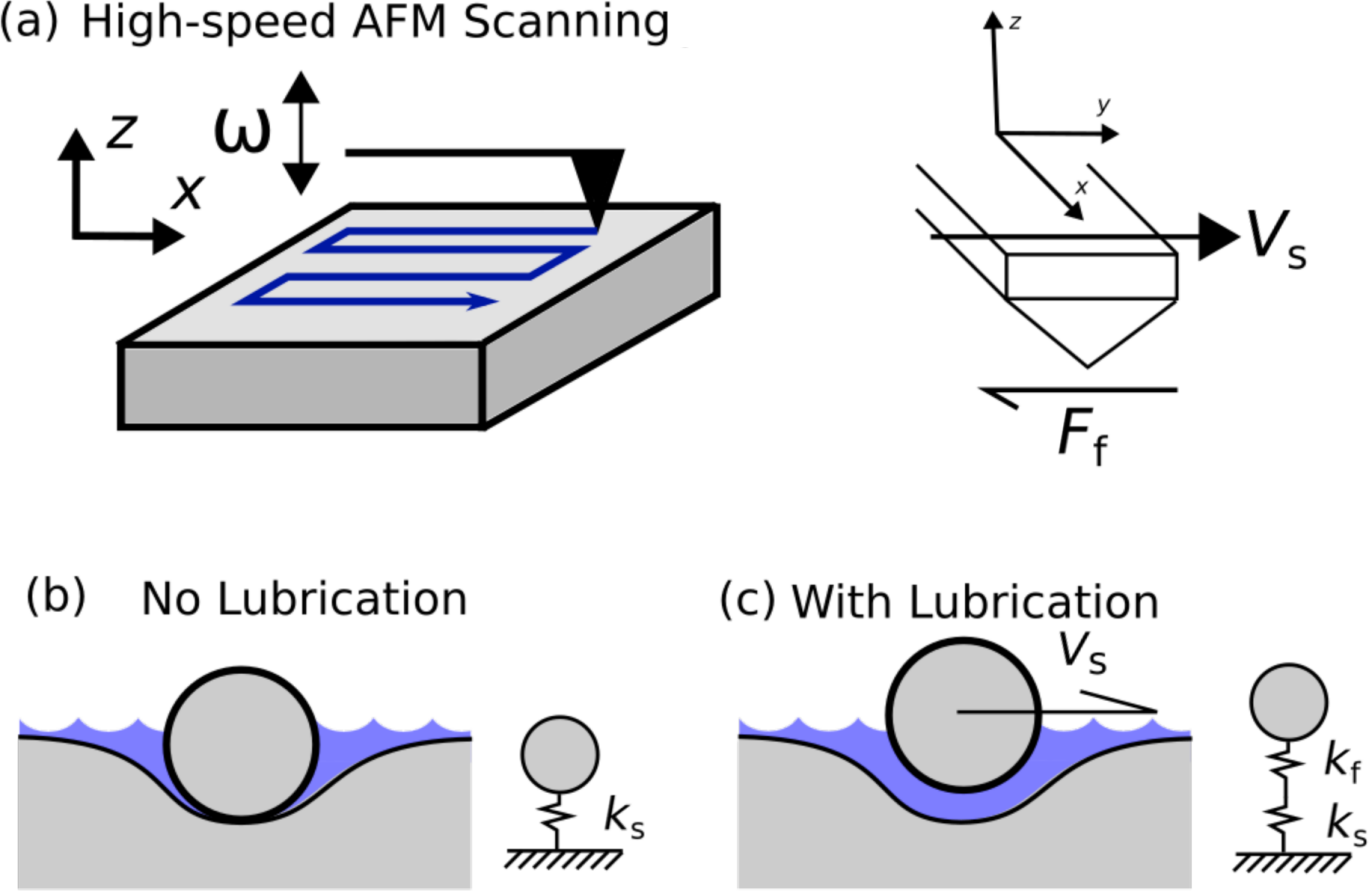
Hydrodynamic lubrication phenomenon. (a) The AFM tip is in intimate contact with the sample surface oscillating with a frequency ω, scanned at a fixed velocity *V*_s_, and experiencing frictional forces *F*_f_. (b) The stiffness detected by the AFM probe is represented solely by the sample stiffness *k*_s_. (c) At a critical velocity, a thin film of supportive water forms between the AFM tip and sample. The stiffness detected by the AFM probe is now represented by the series summation of the water film stiffness *k*_f_ and the sample stiffness *k*_s_.

In air, a native adsorbed layer of water exists on all surfaces. This layer, in some cases, is several nanometers thick [16–17] and is usually studied by measuring adhesive forces between the AFM tip and sample. Capillary necking between the adsorbed water layer and AFM tip creates additional adhesive forces that can be easily measured with a typical AFM system. These measurements are conducted using force pull-off techniques and are not conducted while the AFM tip is dynamically rastered across the sample. Figure 1 depicts the dynamic phenomenon we are investigating.

In Figure 1a, the AFM tip is in contact with the elastic sample oscillating with a frequency ω and is moved across the sample at a fixed velocity *V*_s_. The adsorbed water layer is surrounding the tip–sample junction but does not exist in the contact region. This case, depicted in Figure 1b, represents a situation where the AFM is solely measuring the elastic properties of the sample. The elasticity of the sample has been represented as the linear spring *k*_s_. The AFM tip is moved at a velocity *V*_s_ across the sample when laterally scanning. At a critical speed, we posit that hydrodynamic lift is achieved, and the AFM tip is in sole contact with the adsorbed water layer on the sample. In this situation, depicted in Figure 1c, the measured stiffness is now a series combination of the fluid film stiffness *k*_f_ and the material stiffness *k*_s_. There may also be additional damping effects introduced by the fluid film, which we would like to address in future research studies. For instance, it is known that the modulation of the tip–sample contact has an effect on the friction [18–19]. Furthermore, this effect depends on the scan speed and can bring the system from a stick–slip state to a “steady sliding” state above a critical velocity [18]. It is noted that “a small viscous damping contribution in the tip–sample contact is sufficient enough to suppress stick–slip oscillations” [18]. It may be possible that the thin film acts as a source of viscous damping that allows the system to achieve a “steady sliding” state, above a critical velocity, which may have an effect on the CR measurements.

The hydrodynamic lift force *F* varies approximately as *V*_s_/*h*^3^ for a simple two-dimensional slider model [20], where *V*_s_ is the velocity of the slider and *h* is the fluid gap height. Using this simple model, the hydrodynamic stiffness *k*_f_, which is proportional to ∂*F*/∂*h* (
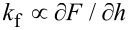
), varies approximately as *V*_s_/*h*^4^. A very small fluid film layer can provide a very large normal stiffness to the AFM tip. Here we have neglected surface roughness, which will provide an upper bound to the fluid stiffness that is experimentally achievable.

**Figure 2:**
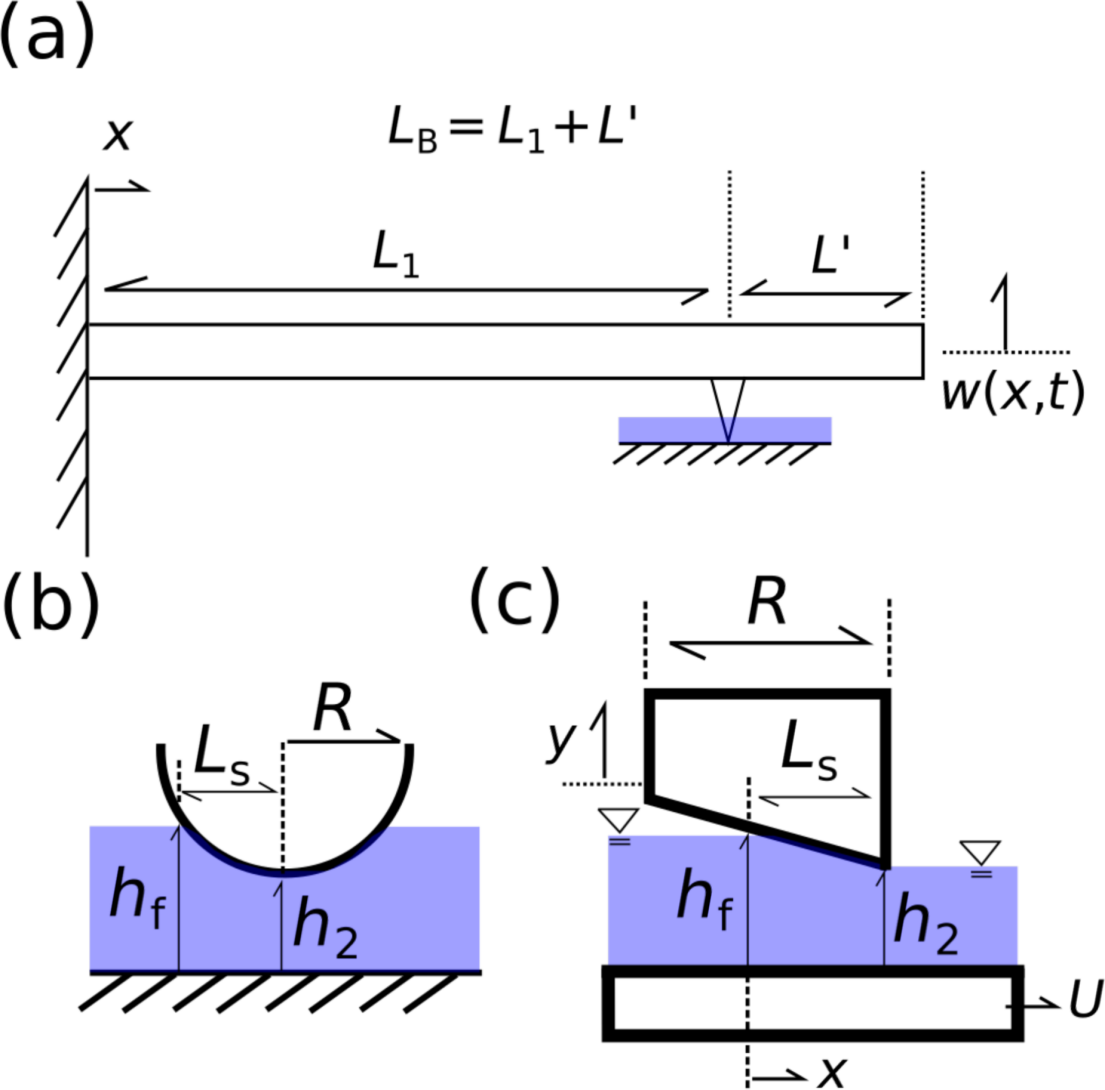
(a) Schematic of the CR system under analysis. The total length of the beam *L*_B_ = *L*_1_ + *L*′ is comprised of the distance from the fixed end of the beam to the tip position given by *L*_1_, and the distance from the tip position to the free end of the beam given by *L*′. (b) Spherical AFM tip submerged in fluid of height *h*_f_ and a distance *h*_2_ from the sample surface. (c) Approximated tip geometry. Stationary inclined slider of length *L*_S_. The sample surface moves at a uniform speed *U* = *V*_S_.

To estimate the vertical lift force and subsequent film stiffness generated by the surface water layer, we utilize a simple slider model with two nonparallel plates. Figure 2b depicts the spherical tip of the AFM probe with radius *R*. We approximate the spherical probe geometry as a stationary inclined plane, as seen in Figure 2c. The sample surface moves at a uniform velocity *U*. Using geometric arguments based on the spherical tip geometry, the length of the slider *L*_S_ is given by


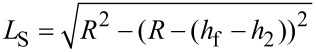


where *h*_f_ is the height of adsorbed fluid layer and *h*_2_ is the distance from the tip of the slider to the sample surface. The width of the slider (normal to the plane of Figure 2c) is given by *W*. It is assumed that *W* = 2*L*_S_ at each instant. We acknowledge that more advanced, geometrically accurate models exist. However, we expect the general trends in the hydrodynamic lift force to remain unchanged.

We begin with the Reynolds lubrication equation for a planar channel given by:

[1]
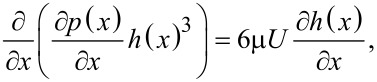


where *p*(*x*) is the pressure in the channel, *h* is the gap height, μ is the fluid viscosity, *U* is the velocity of the bottom plate, and *x* is the horizontal measure of the distance from the beginning of the channel. We assume that the gap height *h* varies linearly and is given by *h*(*x*) = *h*_f_ − ε*x*, where ε = (*h*_f_ − *h*_2_)/*L*_S_ is the angle of the slider.

Solving for the pressure distribution in the channel by integrating Equation 1 with respect to *x* and using the boundary conditions *p*(0) = *p*(*L*_S_) = *p*_∞_, we obtain:

[2]
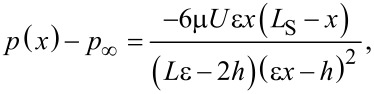


where *p*_∞_ is the ambient fluid pressure outside of the channel. We assume that 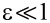 and that *h*_f_ ≈ *h*_2_ ≈ *h* and that 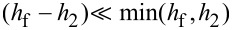. With these assumptions, the ε terms in the denominator of Equation 2 can be neglected. Integrating across the length of the channel, we obtain the vertical lift force *F*:

[3]
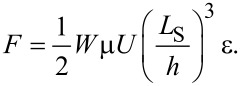


The fluid film stiffness is then given by:

[4]
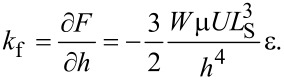


In order to measure the sample stiffness using CR, we use a combination of measured in-contact resonance frequencies. The cantilever beam is modeled as a two-span beam using the Euler–Bernoulli equation [21]. The total length of the beam *L*_B_ = *L*_1_ + *L*′ is comprised of the distance from the fixed end of the beam to the tip position given by *L*_1_, and the distance from the tip position to the free end of the beam given by *L*′ as seen in Figure 2a. We define the tip location parameter 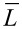 such that 
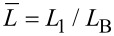
. The beam equation is solved, and a characteristic equation relating the *n*-th non-dimensional contact wavenumbers 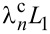 of the beam to the normalized contact stiffness α and the tip parameter 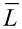 is generated (see Rabe et al. [22]). The normalized contact stiffness, in the absence of a fluid layer, is defined as α = *k*_s_/*k*_c_, where *k*_s_ is the sample stiffness and *k*_c_ is the static cantilever stiffness (
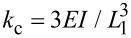
). The characteristic equation has the form 
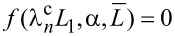
. Using the measured in-contact frequencies, we can calculate the non-dimensional wavenumbers using the relation


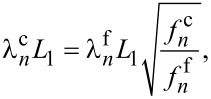


where 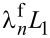 are the known non-dimensional wavenumbers for a freely vibrating cantilevered beam (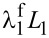 = 1.8751, 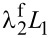 = 4.6941, 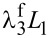 = 7.8548), 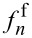 are the measured free frequencies of the AFM cantilever, and 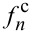 are the measured in-contact frequencies of the AFM cantilever. The tip parameter 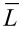 is calculated using the lowest-speed contact frequency pair at the highest set point load. Once 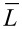 is calculated, α can be calculated for a given contact frequency. The sample stiffness has a direct influence on the error that is introduced by the fluid-film phenomenon. When a gap is formed, the effective stiffness (which is what the AFM system measures) of the contact becomes


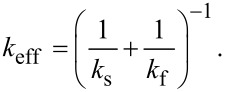


The presence of the fluid stiffness *k*_f_ introduces an error in the AFM measurement of the sample stiffness. This error is dependent on the relative magnitudes between *k*_s_ and *k*_f_.

## Experimental

A sample of high-grade mica was mounted to a steel puck using cyanoacrylate. A razor blade was then used to cleave the sample, leaving behind a pristine sample surface. The sample was placed in a closed AFM flow-cell with an integrated relative humidity sensor. A cantilever with spring constant *k*_L_ = 1.7 ± 0.2 N/m and first free resonance 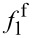 = 61.85 ± 0.1 kHz was mounted to a custom cantilever holder with an integrated highly-damped piezo actuator. The cantilever was chosen to maximize force control while still maintaining resolvable frequency sensitivity. Additionally, the frequency bandwidth of the AFM (2 MHz) and the drive and detection sensitivity of the third eigenmode placed further constraints on the selection of the cantilever. A gas mixing apparatus was used to mix streams of dry and saturated N_2_ to achieve various levels of relative humidity in the cell. The cantilever was brought into contact with the sample at 100 nN and 300 nN. The NIST SPRITE circuit [23] was used to drive the cantilever-holder actuator and to excite and track the first and second CR frequencies while the scan velocity was randomly varied between 0.05 and 100 μm/s. The experiments were repeated on a sample of highly oriented pyrolytic graphite (HOPG).

In this set of experiments, we have controlled for the effect of tip wear by rigorously pre-wearing the AFM tip and randomizing the order of data collection. Tip wear can significantly alter the geometry of a new AFM tip and thus the measured CR frequency. These wear effects must be accurately accounted for. It is well-known that the majority of tip wear happens early in the usage cycle of the microcantilever when the tip is pristine and extremely sharp [24–25]. By pre-wearing the tip, we ensure that large scale geometric evolution of the tip does not occur. Additionally, to control for the effect of wear over time, the experiments were conducted in a random order. Randomizing the testing matrix ensures that cumulative wearing effects will not heavily bias the data.

## Results and Discussion

**Figure 3:**
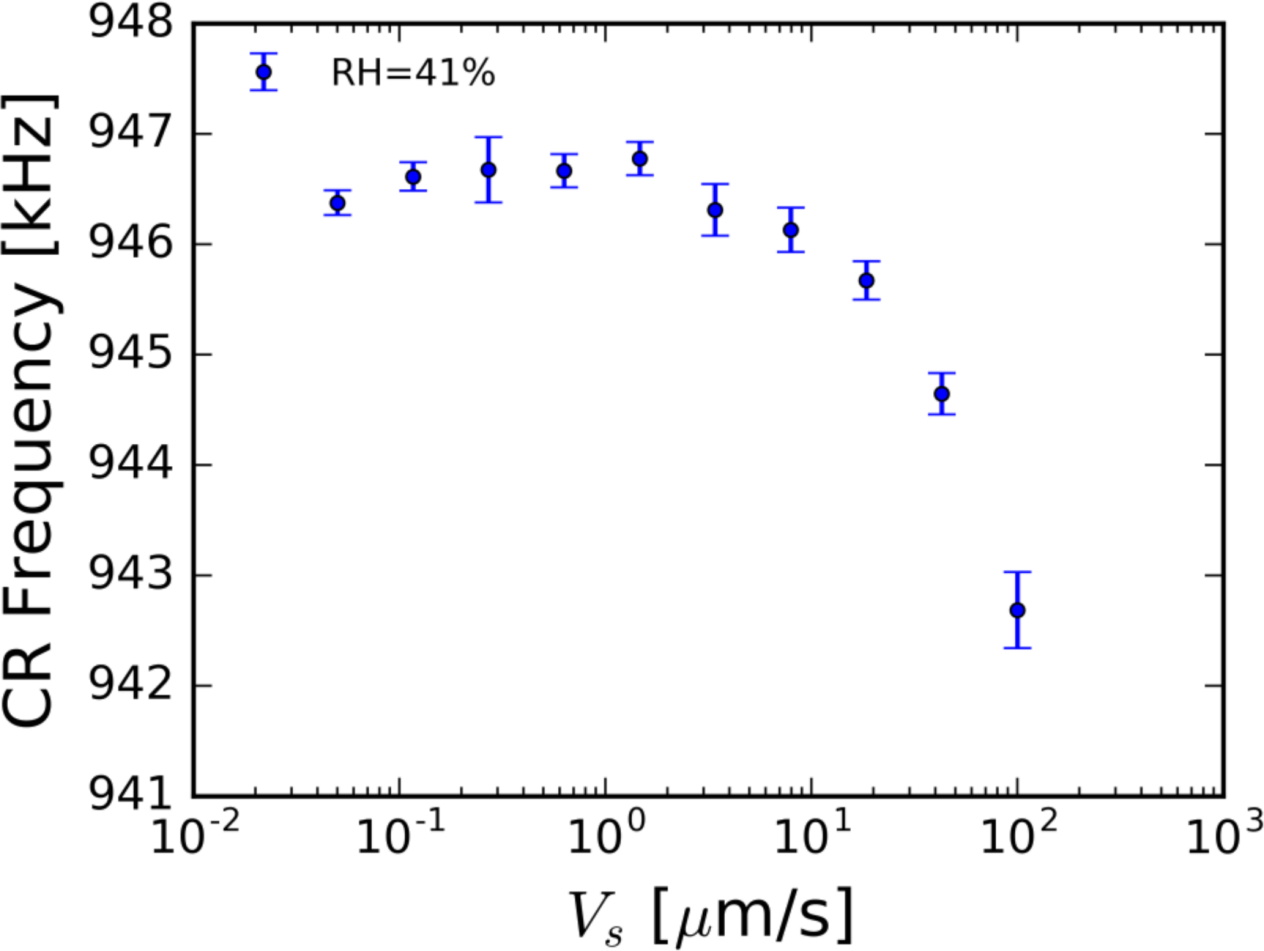
The measured in-contact resonance frequency of the second mode of an AFM cantilever as a function of the dynamic scan speed on a mica surface at 100 nN force set-point, 41% relative humidity, and a scan angle of 90°. The measured in-contact frequency is clearly affected by scan speed. Additionally, this phenomenon is observed at a speed two orders of magnitude lower than reported by Picco and co-workers [15]. Error bars represent one standard deviation from the mean.

Figure 3 shows the measured CR frequency of the second bending mode of the cantilever as a function of the scan speed. The cantilever is indented into a mica surface with a force of 100 nN in an environment with a relative humidity (RH) of 41% and scanned in a direction orthogonal to the long axis of the cantilever (scan angle of 90°). The resulting in-contact natural frequencies of the cantilever are measured while the tip is moved across the sample surface at various velocities. It is clear from Figure 3 that there is indeed a scan speed-dependent phenomenon occurring. Additionally, this phenomenon is evident at scan speeds two orders of magnitude lower than reported by Picco and co-workers [15]. Though the effect of this phenomenon appears small at low scan speeds, we believe it will increase with higher scan speeds. Conventional theories, in which scan velocities are ignored, cannot account for this behavior.

**Figure 4:**
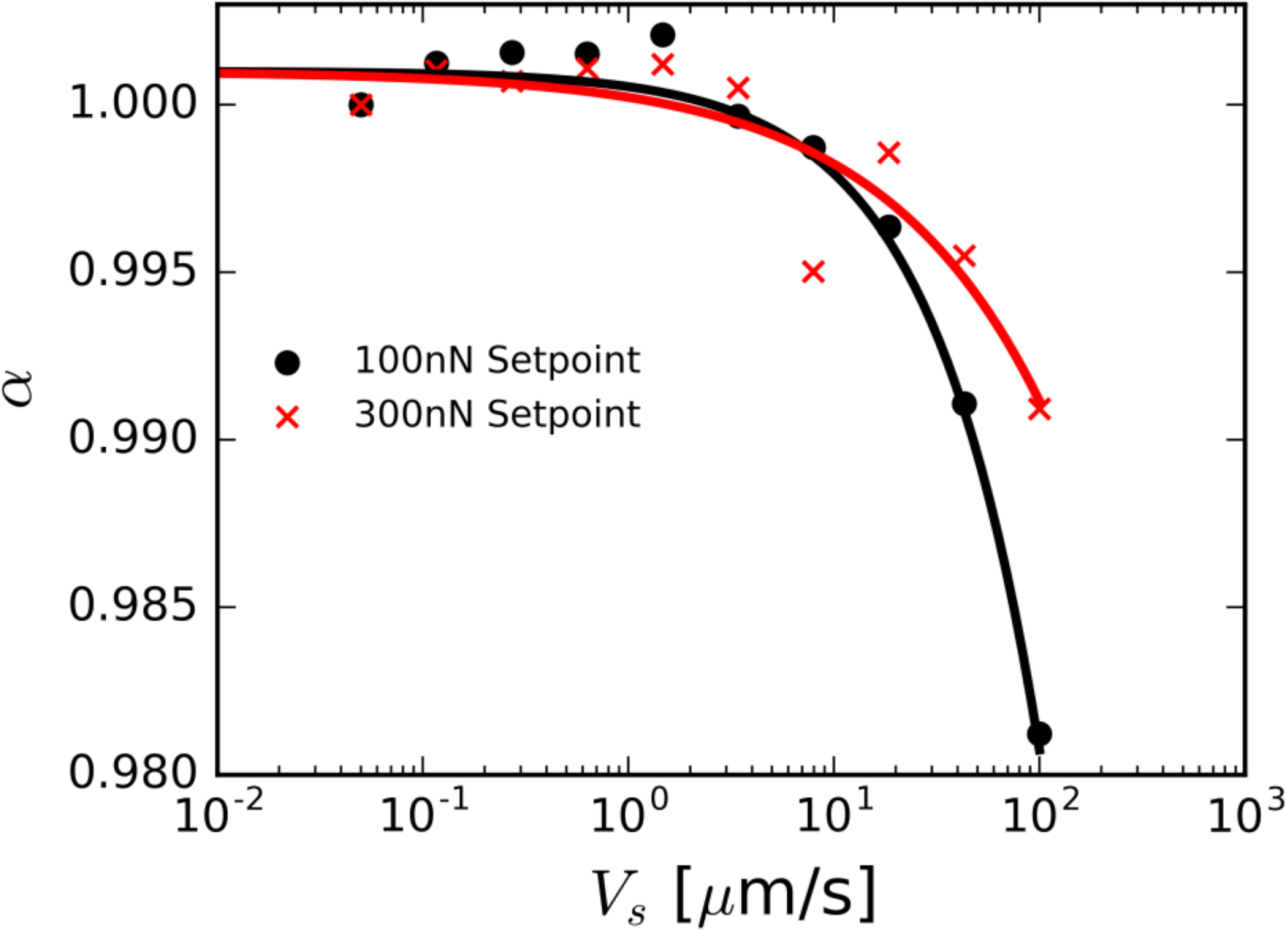
Measured normalized sample contact stiffness as a function of the scan speed. The red data indicates a force set-point of 300 nN. The black data indicates a force set-point of 100 nN.

Using the value calculated for α at zero scan speed and the value calculated for *k*_c_, we can estimate the sample stiffness parameter *k*_s_ for our mica sample. It was found that the stiffness for mica, for our experimental parameters, is approximately 350 N/m. In Figure 4, we show the measured normalized sample contact stiffness α as a function of the scan speed *V*_s_ calculated using data from the 1st and 2nd in-contact natural frequencies measured on mica at 41% relative humidity and a scan angle of 90°. The red line indicates a force set-point of 300 nN. The black line indicates a force set-point of 100 nN. In Figure 4, we see the measured stiffness of the sample decreasing with scan speed above a critical speed. This effect is more dramatic for lower set point forces. We posit that this is due to a delayed hydrodynamic lift phenomenon. For a larger contact force set-point, a higher scan velocity must be reached in order to generate a stable fluid film gap capable of supporting the cantilever tip.

**Figure 5:**
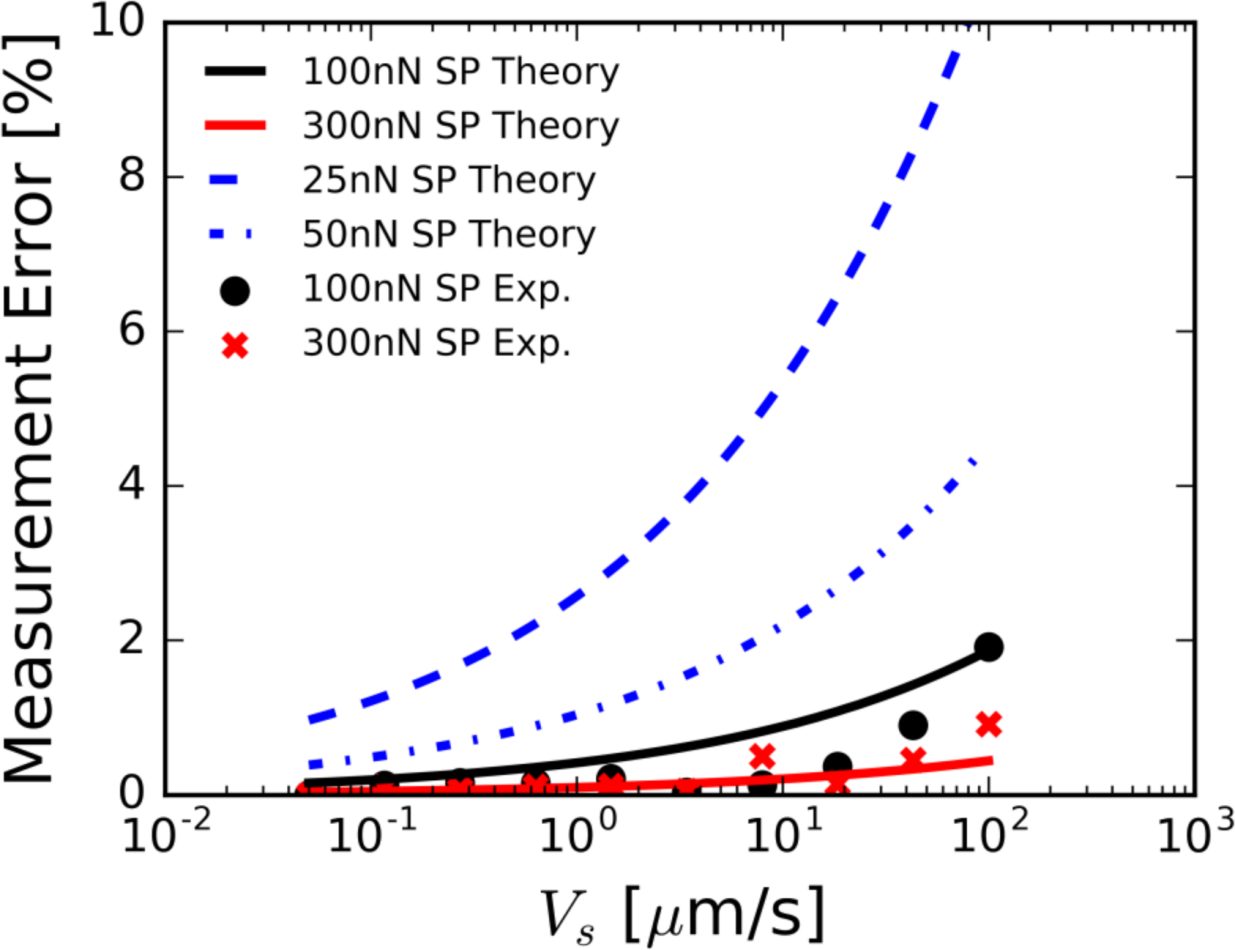
Measurement error introduced by the scan speed phenomenon. Black circles and red crosses represent the percent error in measured sample stiffness on mica as compared with the stationary measurement (for 100 and 300 nN force set-point (SP), respectively). The black and red lines represent the percent error calculated by the lubrication theory given in Equation 3 and Equation 4. The percent error was calculated using the measured stationary sample stiffness of mica (*k*_s_ = 350 N/m) and the equation for the effective sample stiffness *k*_eff_. The blue dashed line represents the theoretical percent error for a force set-point of 25 nN. The blue dashed and dotted line represents the theoretical percent error for a force set-point of 50 nN.

Using Equation 3, Equation 4, the experimental scan speeds, and the experimental force set-points, we estimate the fluid forces on the AFM tip. We have used the parameter values μ = 8.94 × 10^−4^ Pa·s for the viscosity of water, *R* = 150 nm for the radius of the AFM tip, and *h*_f_ = 1 nm for the fluid film height on the sample. The fluid film height has been chosen to represent reported values in the literature for similar experimental conditions [26–27]. For each contact force set-point and scan speed, Equation 3 is solved for the gap height *h*_2_. Here we have replaced *h* with *h*_2_, following our aforementioned assumptions. The fluid film stiffness can be computed using Equation 4 once *h*_2_, the distance the tip of the slider is from the sample, is calculated. The theoretical percent error introduced by the fluid film stiffness is then calculated using the experimentally obtained sample stiffness for mica (*k*_s_ = 350 N/m) measured at zero scan speed and the equation for the effective sample stiffness *k*_eff_. The experimental percent error in the stiffness measurement is calculated using the difference between the measured sample stiffness at zero scan speed and at non-zero scan speeds. Figure 5 shows both the experimental and theoretical values for the percent error introduced. For the given parameters, the model qualitatively captures the behavior of the error growth, despite the simplicity of the model. Additionally, we see that the magnitude of the error is increased as the force set-point is decreased for a given speed. This behavior matches the experimentally observed trends. We note that the gap heights calculated using the assumed theory are well below the applicable range for a fluid described by continuum theory. Further research must be performed to develop higher fidelity experimental models.

As stated previously, we believe that the presence of a thin film of water and hydrodynamic lift explains the scan speed-dependent phenomenon observed on our mica sample at 41% RH. Many studies have found that an “ice-like” or “solid-like” layer of structured water is found on hydrophilic surfaces under the right temperature, humidity, and loading conditions [17,26,28–32]. Researchers using molecular dynamic (MD) simulations have also found that “at the monolayer coverage, water forms a 2-D H-bonded network in an epitaxial relationship with the mica lattice” [17]. The first complete layer of water is thought to be approximately 1 nm in thickness and formed at approximately 40% RH on mica [26–27]. The water film was found to have elastic, viscous, and energy dissipation properties that changed when the driving amplitude was varied [30,33–34]. Hofbauer et al. found that “the mechanical stress exerted by the vibrating AFM tip leads to periodic compression and decompression of the underlying molecular lattice” [35]. The magnitude of the applied force, the rate of change of the applied force, and the tip–sample gap were also found to affect the properties of the thin film [17,33,36–37]. Antognozzi et al. [28] found that when a fluid is highly confined, a manifestation of elastic behavior is produced, and Li et al. [27] found that the viscosity of nanoconfined water increases with increasing confinement. All of these findings suggest that the dynamic properties of the thin film are affected by external loading. Many researchers have reported an increase in the viscosity of the confined water film of many orders of magnitude compared with bulk properties [17,27,31,36–37]. With a greater understanding of the mechanical behavior and properties of the thin water film under different loading conditions, we may be able to enhance our simple slider model by using higher-order models that extend beyond the continuum assumptions for the confined fluid.

**Figure 6:**
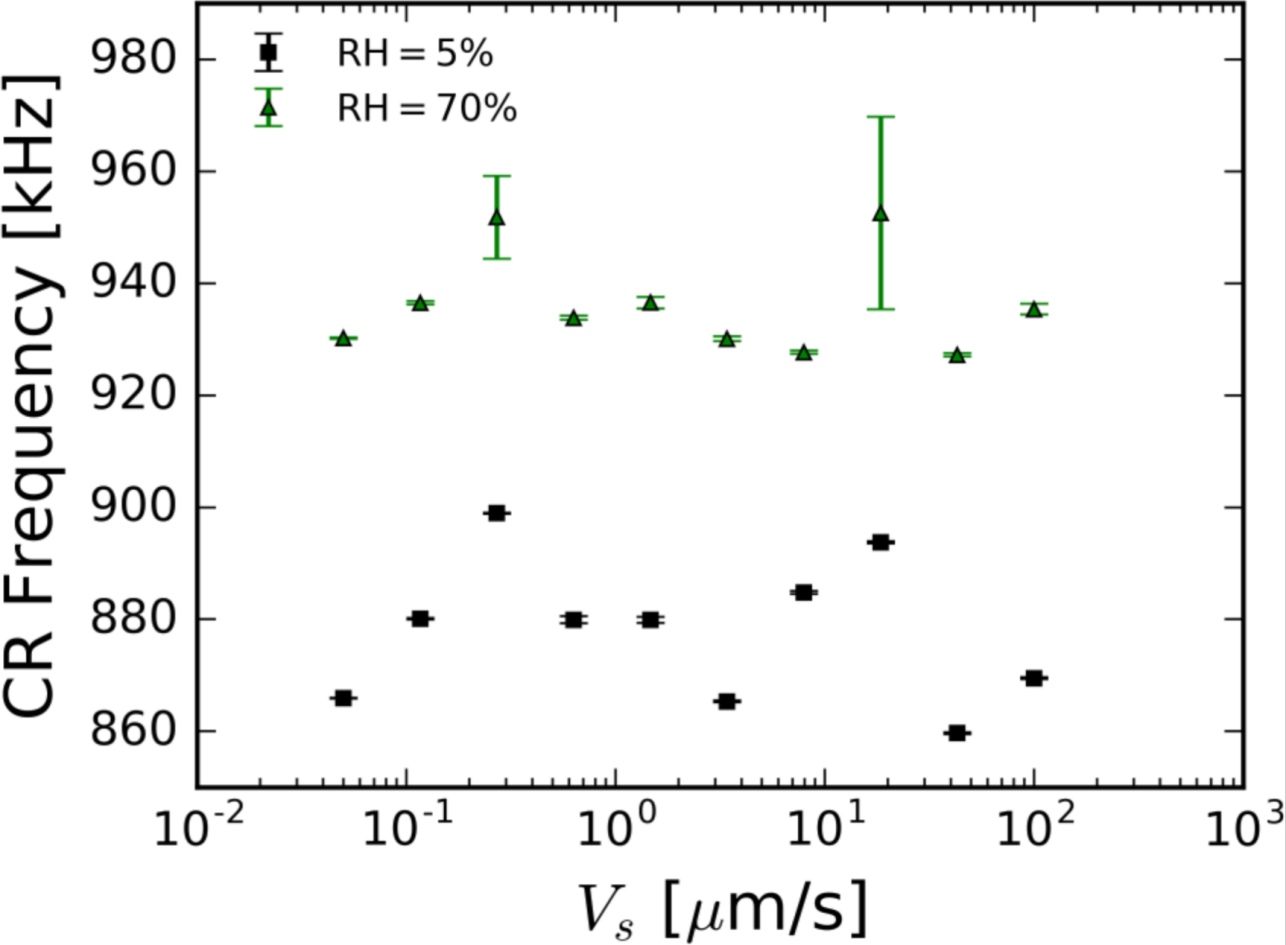
The measured in-contact resonance frequencies of an AFM cantilever as functions of the dynamic scan speed on a mica surface at 100 nN force set-point and 5% and 70% relative humidity. No scan speed-related phenomena are observed. Error bars represent one standard deviation from the mean.

**Figure 7:**
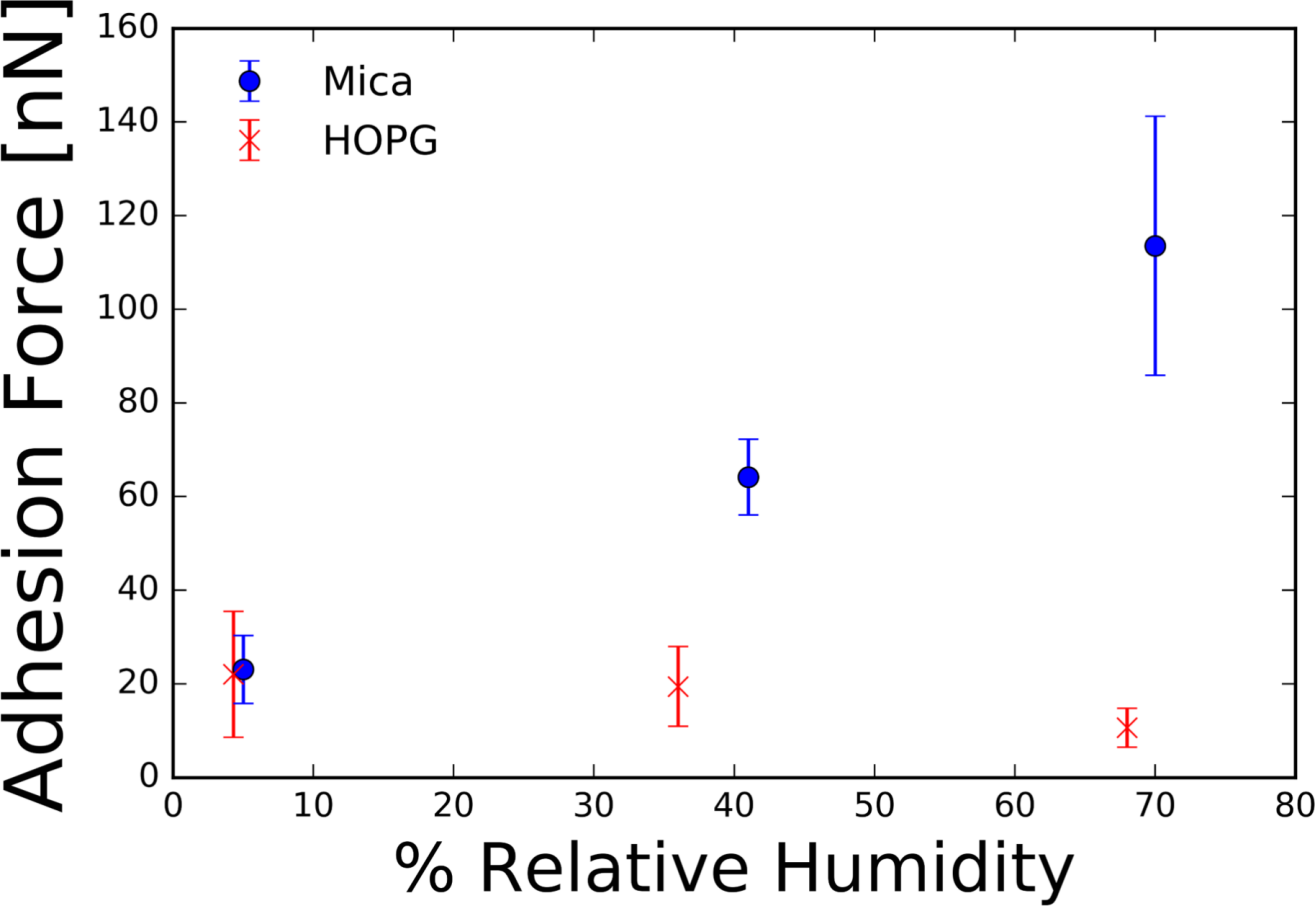
Average measured adhesion force on mica and HOPG samples at various values of relative humidity. The hydrophilic mica shows an increasing adhesion force with increasing humidity while the hydrophobic HOPG shows a nearly constant adhesion force as a function of relative humidity. Error bars represent one standard deviation from the mean.

The scan speed phenomenon was not observed on mica at 5% RH or on HOPG at 4.3% RH. We believe that the absence of the observed phenomenon is due to the lack of formation of a thin water layer on the surfaces of the samples at low humidity. Figure 6 shows the recorded contact-resonance frequencies on mica under low- and high-humidity conditions. Figure 7 shows the measured adhesion force as a function of the relative humidity for different RH values for both mica and HOPG. On the hydrophilic mica sample, a distinct increase of measured adhesion forces is apparent with increasing relative humidity. This suggests the growth of the thin water film on mica with increasing relative humidity. The observed behavior of the adhesion force for hydrophilic and hydrophobic surfaces matches results reported by Bhushan and co-workers [38].

The scan speed phenomenon was not observed on HOPG at 36% or 70% RH. Goertz et al. [37] found that the viscous interfacial water film did not exist when the hydrophilicity of their oxide-terminated silicon surface was degraded, changing its behavior to hydrophobic. From this observation, they proposed that the surface must be hydrophilic in order to form a viscous interphase water film. Therefore, it is plausible that a thin, highly ordered, viscous water film cannot form on the hydrophobic HOPG sample. Additionally, nanobubbles on the sample surface may also affect the formation of a thin, highly ordered, viscous water film. Maali et al. [39] posited that the presence of nanobubbles explains liquid slip at the interface and the long-range attraction between hydrophobic surfaces in water. Maali et al. [39] and Tyrell et al. [40] were able to image nanobubbles in tapping mode and found that the bubbles could be easily moved by the probe tip and could not be imaged in contact mode. The presence of nanobubbles on the hydrophobic HOPG sample may prevent a continuous film from forming.

The scan speed phenomenon is also absent on mica at 70% RH (see Figure 6). Zhao et al. [26] estimated the water film on mica to be approximately 1.5 nm at 70% RH, which suggests the existence of a multi-layered water film. The formation of multiple water layers at higher humidity levels has been seen by Verdaguer and co-workers [17]. The existence of multiple water layers may induce a phenomenological change in the interaction of the AFM tip and sample. Spagnoli et al. [30] posited that additional water layers were unable to form multiple hydrogen bonds with the water molecules in the spontaneously formed ice-like water layer near the substrate. MD simulations by Ou et al. [41] suggest that as the water film grows, the bonds between the water molecules become stronger while the bonds of the water and mica sample become weaker. The fact that the ice-like first layer is still present at higher humidity but the scan speed phenomenon was not observed in the regime suggests that the additional water layers have changed the dynamics of the tip–sample interaction. Furthermore, higher adhesion forces found on mica at 70% RH might change the threshold speed needed to achieve hydrodynamic lift.

## Conclusion

This work has shown the existence of a scan speed-related phenomenon in contact mode AFM. Specifically, this phenomenon was measured using contact resonance spectroscopy. Above a critical speed, a monotonic decrease in the contact resonance frequencies was observed with increasing scan speed. This phenomenon was explained using hydrodynamic theory. Further research must be conducted to study the effect that a thin, highly ordered, viscous water layer has on the dynamics of the tip–sample contact at various relative humidity and on hydrophilic and hydrophobic samples.
